# Aspirin resistance in pregnancy is associated with reduced interleukin-2 (IL-2) concentrations in maternal serum: Implications for aspirin prophylaxis for preeclampsia

**DOI:** 10.1016/j.preghy.2024.101131

**Published:** 2024-06-07

**Authors:** Fernando Hernandez, Hector Chavez, Sophie L. Goemans, Yeva Kirakosyan, Carolina Diaz Luevano, Dana Canfield, Louise C. Laurent, Marni Jacobs, Doug Woelkers, Maryam Tarsa, Cynthia Gyamfi-Bannerman, Kathleen M. Fisch

**Affiliations:** Department of Obstetrics, Gynecology & Reproductive Sciences, University of California, San Diego, La Jolla, CA, USA

**Keywords:** Preeclampsia, Low dose aspirin, Aspirin resistance, Circulating biomarkers

## Abstract

**Objectives:**

To evaluate the impact of aspirin resistance on the incidence of preeclampsia and maternal serum biomarker levels in pregnant individuals at high-risk of preeclampsia receiving low dose aspirin (LDA).

**Study design:**

We performed a secondary analysis of a randomized, placebo-controlled trial of LDA (60 mg daily) for preeclampsia prevention in high-risk individuals (N = 524) on pregnancy outcomes and concentrations of PLGF, IL-2, IL-6, thromboxane B2 (TXB_2_), sTNF-R1 and sTNF-R2 from maternal serum.

**Main outcome measures:**

LDA-resistant individuals were defined as those having a TXB_2_ concentration >10 ng/ml or <75 % reduction in concentration at 24–28 weeks after LDA administration. Comparisons of outcomes were performed using a Fisher’s Exact Test. Mean concentrations of maternal serum biomarkers were compared using a Student’s *t*-test. Pearson correlation was calculated for all pairwise biomarkers. Longitudinal analysis across gestation was performed using linear mixed-effects models accounting for repeated measures and including BMI and maternal age as covariates.

**Results:**

We classified 60/271 (22.1 %) individuals as LDA-resistant, 179/271 (66.1 %) as LDA-sensitive, and 32/271 (11.8 %) as non-adherent. The prevalence of preeclampsia was not significantly different between the LDA and placebo groups (OR = 1.43 (0.99–2.28), p-value = 0.12) nor between LDA-sensitive and LDA-resistant individuals (OR = 1.27 (0.61–2.8), p-value = 0.60). Mean maternal serum IL-2 concentrations were significantly lower in LDA-resistant individuals relative to LDA-sensitive individuals (FDR < 0.05).

**Conclusions:**

These results suggest a potential role for IL-2 in the development of preeclampsia modulated by an individuals’ response to aspirin, presenting an opportunity to optimize aspirin prophylaxis on an individual level to reduce the incidence of preeclampsia.

## 1. Introduction

Aspirin (acetylsalicylic acid) is one of the oldest and most commonly used medications in the United States, due, in part, to its observed positive effects on cardiovascular health [1,2]. Low dose aspirin (LDA) has been demonstrated to decrease the incidence of preeclampsia in several large clinical trials [3–5], although some demonstrate only a modest decrease that is not statistically significant [6–9]. One of the first major trials to evaluate LDA for prevention of preeclampsia included normotensive individuals at 28 weeks gestation thought to be at risk for preeclampsia due to blood pressure response to angiotensin II infusions [10]. The individuals who were randomized to 60 mg of aspirin were 83 % less likely to develop preeclampsia. The CLASP trial later performed a randomized trial of 60 mg aspirin daily in 9,364 pregnant individuals, which found a 12 % reduction in the incidence of preeclampsia, although it was not statistically different from placebo [7]. However, the CLASP trial did find a statistically significant reduction in the likelihood of preterm delivery [7]. A recent systematic review done on behalf of the US Preventative Services Task Force (USPSTF) reviewed 23 randomized clinical trials where LDA was used to prevent preeclampsia [11]. The authors found that aspirin use was associated with a lower risk of preeclampsia (pooled relative risk [RR], 0.85 [95 % CI, 0.75–0.95]), perinatal mortality (pooled RR, 0.79 [95 % CI, 0.66–0.96]), and preterm birth (pooled RR, 0.80 [95 % CI, 0.67–0.95]), with no increase in bleeding-related complications, such as postpartum haemorrhage [11].

LDA (81 mg) is recommended after 12 weeks gestation in pregnant individuals at high risk of preeclampsia or with more than one moderate-risk factor by the USPSTF [11], with similar recommendations by the American College of Obstetricians and Gynecologists (ACOG) and the Society for Maternal-Fetal Medicine [12]. Yet, despite the demonstrated benefits of LDA prophylaxis at the population level, aspirin resistance has been described both within and outside of pregnancy [13,14]. Current national guidelines in the United States place a large number of at-risk pregnant people on LDA [12], although aspirin resistance decreases the potential benefit of this therapy [13,14].

Aspirin resistance, defined using laboratory indices reflective of insufficient platelet inhibition activity [15], is observed in 17–39 % of pregnant individuals, and it has been associated with a higher incidence of preeclampsia despite aspirin administration [14,16–19]. In addition, pregnant individuals with inadequate adherence to an aspirin regimen (<90 %) have been demonstrated to have higher rates of preeclampsia, intrauterine growth restriction, preterm delivery and an increased need for antenatal antihypertensive medications [20]. We hypothesize that aspirin resistant individuals would have a higher incidence of preeclampsia and increased inflammatory cytokine levels in maternal serum. In this study, we investigated clinical outcomes in defined aspirin resistant and nonadherent individuals by maternal serum thromboxane B (TXB_2_) measurements to identify maternal serum biomarker signatures associated with aspirin resistance in preeclampsia.

## 2. Materials and methods

### 2.1. Study cohort

We performed a secondary analysis of a double-blinded, randomized, placebo-controlled trial of LDA (60 mg daily) for preeclampsia prevention in individuals at high risk for preeclampsia (N = 2,539) [6]. Individuals at high risk for preeclampsia were recruited and enrolled between 13 and 26 weeks of gestation and randomized to receive either low-dose aspirin (60 mg daily) or placebo to study the primary outcome of preeclampsia diagnosis. This study was performed as part of the Institute of Child Health and Human Development Maternal Fetal Medicine Unit Network across 13 centers and anonymized data were made publicly available at the Institute of Child Health and Human Development Data and Specimen Hub. Subjects provided written informed consent before study participation and all studies were approved and monitored by each participating institution’s institutional review board.

### 2.2. Maternal serum sample biomarker concentrations

Blood samples were drawn into EDTA-containing tubes at three time points during pregnancy (13–26 weeks at randomization, 24–28 weeks, 34–38 weeks), centrifuged to separate the plasma and serum fractions, and stored at −70 °C for future studies [21]. Stored plasma and serum samples were assayed for placental growth factor (PLGF), interleukin-2 (IL-2), interleukin-6 (IL-6), thromboxane B2 (TXB_2_), soluble tumor necrosis factor receptor 1 (sTNF-R1) and soluble tumor necrosis factor receptor 2 (sTNF-R2) in previous studies [21–24]. For this secondary analysis, we included individuals with complete TXB_2_ measurements at all three time points, complete body mass index (BMI) data and complete biomarker concentration measurements at all three timepoints for PLGF, IL-2, sTNF-R1 and sTNF-R2 (N = 524).

### 2.3. Clinical outcome, gestational age and aspirin resistance group definitions

**Fig. 1.**
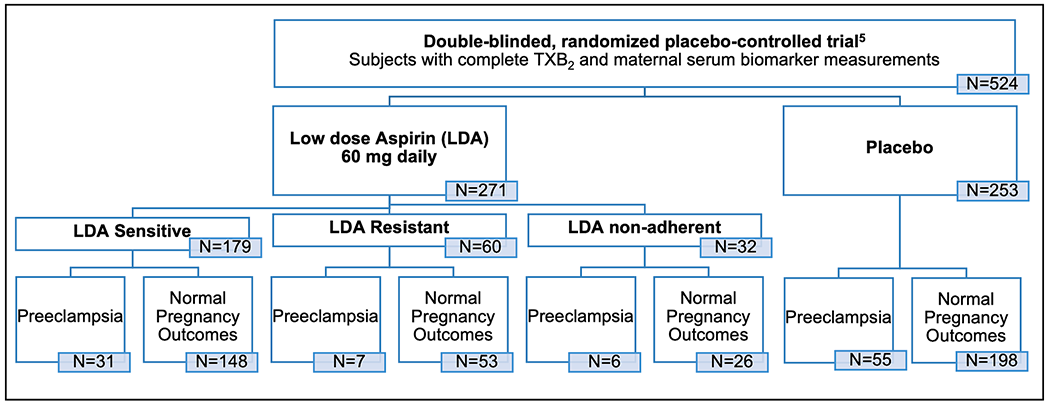
Overview of the study design outlining clinical outcomes and aspirin resistance groups.

Clinical outcomes were separated into six groups by aspirin exposure: placebo with preeclampsia, placebo normal pregnancy outcome, LDA with preeclampsia, LDA normal pregnancy outcome, No LDA with preeclampsia, and No LDA normal pregnancy outcome (Fig. 1). Within the LDA exposed groups, we defined LDA sensitivity as a TXB_2_ concentration <10 ng/ml or >75 % reduction in concentration at 24–28 weeks gestation after aspirin administration. Aspirin resistance groups included: LDA-resistant (TXB_2_ concentration >10 ng/ml or <75 % reduction in concentration at 24–28 weeks gestation after aspirin administration); LDA-non-adherent (demonstrated LDA sensitivity at 24–28 weeks with TXB_2_ concentrations >10 ng/ml at 34–38 weeks); and LDA-sensitive (demonstrated LDA sensitivity at both 24–28 weeks and 34–38 weeks). Estimated gestational age (GA) at each time point was calculated by adding an offset at each timepoint to GA at randomization to create a continuous variable assuming eight weeks between biospecimen acquisition for all subjects (Time 1: GA in days at randomization; Time 2: GA + 56 days; Time 3: GA + 112 days).

### 2.4. Statistical analysis

Comparisons of categorical outcomes by group were performed using a Fisher’s Exact Test or Kruskal-Wallis rank sum test for continuous variables. Mean concentrations of selected maternal biomarkers were analyzed using a pairwise Student’s *t*-test within clinical response groups and aspirin resistance groups at each timepoint. Adjustment for multiple testing was performed using Benjamini & Hochberg (FDR) correction [25]. Statistical significance for pairwise tests was set at FDR < 0.05. Pearson correlation was calculated for all pairwise biomarkers. Longitudinal analysis across gestation was performed using linear mixed-effects models accounting for repeated measures and including body mass index (BMI) and maternal age as covariates on center scaled [26] log2-biomarker concentrations and GA using lmerTest v3.1–3 [27]. Statistical significance for longitudinal analyses was set at p-value < 0.05. All analyses were performed using the R Statistical Programming Language (v4.1.3) using the R packages lmerTest v3.1–3, ggplot2 v3.4.0, and stats v.4.1.3.

## 3. Results

We classified 60/271 (22.1 %) individuals as LDA-resistant, 179/271 (66.1 %) as LDA-sensitive and 32/271 (11.8 %) as non-adherent. Baseline demographic characteristics were not significantly different between LDA and placebo and LDA resistance groups (Table 1). The prevalence of preeclampsia was lower in the LDA group compared to the placebo group, but was not significantly reduced, consistent with the original study [6] (OR = 1.43, 95 % CI 0.99–2.28, p-value = 0.12). Within the LDA subjects, the prevalence of preeclampsia did not differ between LDA sensitive and LDA resistant individuals (OR = 1.27, 0.61–2.8, p-value = 0.60).

Mean maternal serum IL-2 concentrations were significantly lower in LDA-resistant individuals relative to LDA-sensitive individuals after LDA administration among normal pregnancy outcomes (FDR < 0.05; Table 2; Supplementary Fig. 1) and overall (FDR < 0.05; Table 3). Mean concentrations of all biomarkers did not significantly differ between LDA resistance groups within individuals that developed preeclampsia, although LDA-sensitive individuals had higher levels of IL-2 compared to those that were LDA-resistant (Table 3). IL-2 concentrations were nominally elevated in preeclampsia relative to subjects with normal pregnancy outcomes in the placebo group (FDR = 0.112; nominal p-value = 0.036; Table 4; Supplementary Fig. 1). Within the placebo group, IL-6, sTNF-R1 and sTNF-R2 mean concentrations were higher in preeclampsia at 34–38 weeks (FDR < 0.05) and PLGF mean concentrations were lower in preeclampsia at 24–28 and 34–28 weeks (FDR < 0.05; Table 4; Supplementary Figs. 2–3).

**Fig. 2.**
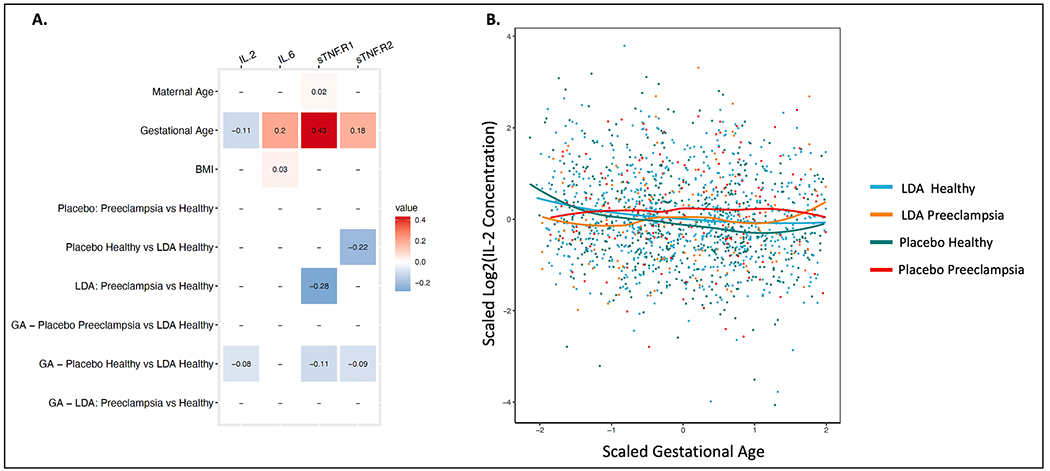
Longitudinal analysis of biomarker concentrations across gestation in LDA and Placebo groups stratified by clinical outcome. **A.** Linear mixed-effects model coefficients for significant terms (p-value < 0.05). **B.** Scatterplot of scaled log2(IL-2) concentration across scaled gestation.

**Fig. 3.**
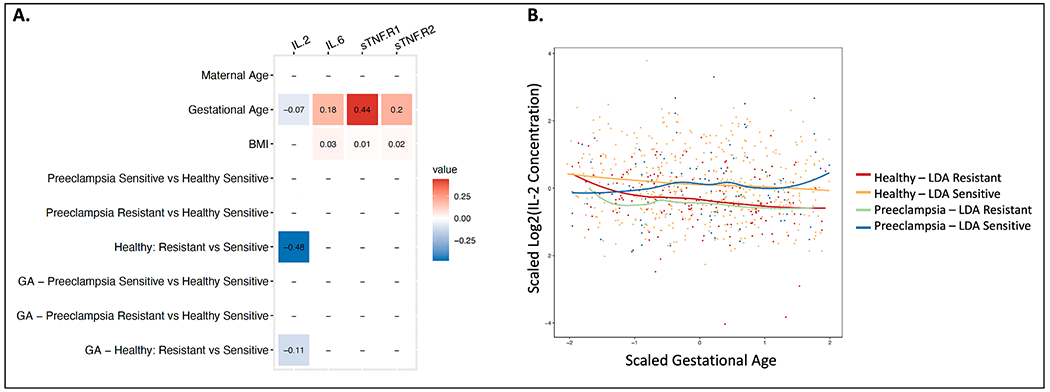
Longitudinal analysis of biomarker concentrations across gestation in LDA clinical outcomes stratified by aspirin resistance. **A.** Linear mixed-effects model coefficients for significant terms (p-value < 0.05). **B.** Scatterplot of scaled log2(IL-2) concentration across scaled gestation.

Longitudinal analysis of additional maternal serum biomarker concentrations across gestation stratified by clinical outcome identified an increase in IL-6, sTNF-R1 and sTNF-R2 with gestational age (p-value < 0.05) and a decrease in IL-2 concentrations with gestational age (Fig. 2A; p-value < 0.05). A significantly different relationship with gestational age was observed within subjects with normal pregnancy outcomes between LDA and Placebo for IL-2 (coefficient = −0.08; p-value < 0.001; Fig. 2A–B; Supplementary Fig. 1), sTNF-R1(coefficient = −0.11; p-value < 0.001; Fig. 2A; Supplementary Fig. 2) and sTNF-R2 (coefficient = −0.09; p-value < 0.001; Fig. 2A; Supplementary Fig. 3). Longitudinal analysis of biomarker concentrations across gestation in LDA clinical outcomes stratified by LDA resistance group revealed a significant decrease in IL-2 concentrations in LDA-resistant relative to LDA-sensitive individuals within the group of subjects with normal pregnancy outcomes (coefficient = −0.48; p-value < 0.05; Fig. 3A–B) and a significantly different relationship with gestational age (coefficient = −0.11; p-value < 0.05; Fig. 3A–B; Supplementary Fig. 1). Finally, TXB_2_ concentrations were significantly negatively correlated with IL-2 (Corr: −0.073; p-value < 0.05), sTNF-R1 (Corr: −0.26; p-value < 0.001) and sTNF-R2 (Corr: −0.17; p-value < 0.001) (Supplementary Fig. 4).

## 4. Discussion

We conducted a secondary analysis of a randomized placebo-controlled trial to evaluate the impact of aspirin resistance on the incidence of preeclampsia and selected maternal serum biomarker levels in a pregnant population at high-risk of developing preeclampsia receiving low dose aspirin. This study revealed that 22.1 % of pregnant individuals on LDA demonstrated aspirin resistance. This rate of aspirin resistance is comparable to that observed in previously published reports in several populations, ranging from 17-39 % of pregnant individuals [14,16–19]. Baseline demographics were not different between groups. The prevalence of preeclampsia was lower in the LDA group compared to the placebo group but was not significantly reduced, consistent with the findings of the original clinical trial [6]. Gestational age was significantly positively associated with maternal serum concentrations of IL-6, sTNF-R1 and sTNF-R1 and negatively associated with IL-2. This study reveals increased IL-2 concentration in preeclampsia, decreased IL-2 concentrations in LDA-resistance and differential changes of IL-2 throughout gestation based on aspirin resistance.

While full dose aspirin (325 mg daily) inhibits both cyclooxygenase-1 (COX-1) and cyclooxygenase-2 (COX-2), LDA selectively inhibits COX-1 [28]. Selective inhibition of COX-1 which targets thromboxane A_2_, is known to occur at doses < 300 mg, but a debate exists in the literature about the appropriate aspirin dose to mitigate preeclampsia risk [17,29,30] and the role of aspirin resistance [13,14].

Aspirin resistance has several proposed mechanisms including pharmacokinetic type resistance from patient non-compliance, suboptimal dosing or increased platelet turnover [13,31]. Physiological changes during pregnancy can affect aspirin pharmacokinetics. For example, one pharmacokinetic study of LDA during pregnancy demonstrated reduced exposure to the active metabolite – salicylic acid – as a result of increased clearance during pregnancy [32]. Another study observed lower rates of complete inhibition of TXB_2_, a stable metabolite of TXA_2_, in obese individuals in the second and third trimesters [33]. Other proposed mechanisms include pharmacodynamic type resistance due to genetic polymorphisms in COX-1 and other genes involved in thromboxane biosynthesis [13,34–35], or TXA_2_- independent platelet aggregation as a result of exposure of platelets to collagen, increased epinephrine, increased oxidative stress or genetic polymorphisms in the common pathway of platelet aggregation [13,31,36–38]. Aspirin resistance may be overcome by altered dosing, minimizing thromboxane influence or by blocking other pathways of platelet activation or inflammation [13], underscoring the importance of identifying aspirin resistance early in pregnancy.

Both the innate and adaptive immune systems in preeclampsia are activated with increased levels of circulating pro-inflammatory cytokines, which have been shown to induce prostaglandin E_2_ (PGE_2_) biosynthesis in the decidua through the up-regulation of COX-2 [39]. Aspirin may increase trophoblast cytokine release causing reduced cell apoptosis, changes in cell aggregation and fusion to improve trophoblast function [40,41]. It has also been shown that aspirin can bind cellular kinase IKK-B, preventing NF-kB-mediated regulation of gene expression independent of the COX-prostanoid pathway that will impede downstream activation of COX-2 and TNF-α mediated endothelial dysfunction, further dampening the dysregulated inflammatory state of preeclampsia [39]. In addition, LDA has been show to trigger the biosynthesis of endogenous anti-inflammatory 15-epi-lipoxin A_4_ (ATL) and may play a role in the mechanism of action of LDA in preeclampsia prophylaxis [42].

Cell types expressing high levels of COX-2 produce large amounts PGE_2_, which strongly inhibits the production of Th1 cytokines, such as IFN-y and IL-2, and favors type-2 responses [43]. Aspirin has been shown to increase IL-2 production by human peripheral blood lymphocytes [44], which we observe in LDA-sensitive healthy pregnancies but not those that develop preeclampsia as those have already elevated levels of IL-2. IL-2, a pleiotropic cytokine that induces the differentiation of regulatory T cells, drives T cell growth, augments NK cytolytic activity and mediates activation-induced cell death [45–48]. IL-2 is characteristic of Th1-type immunity and has been associated with cell-mediated cytotoxic and inflammatory responses, participating in cellular immunity and the rejection process [49]. The Th1/Th2 balance establishes immune tolerance at the maternal-fetal interface, and a shift in the Th1/Th2 equilibrium towards Th1 dominance has been associated with recurrent miscarriage and other disorders of pregnancy, such as preeclampsia [50–52]. IL-2 and IL-2 receptor maternal serum levels have been demonstrated to be increased in preeclampsia [45,53–56] and decreased throughout gestation in healthy pregnancies [57,58]. In this study, we observe increased IL-2 concentrations in preeclampsia in placebo and decreased IL-2 concentrations in LDA-resistant individuals exposed to LDA. This finding warrants further study, as increased maternal serum IL-2 resulting from LDA administration may exacerbate the pro-inflammatory state in some individuals at high-risk of developing preeclampsia.

### 4.1. Strengths and limitations

The incidence of preeclampsia did not differ between LDA sensitive and LDA resistant individuals in this study, nor between LDA and placebo arms, consistent with the original clinical trial [6]. Thus, our hypothesis that aspirin resistant individuals would have a higher incidence of preeclampsia was not observed in this population. In addition, our hypothesis that aspirin resistant individuals would have increased inflammatory cytokine levels in maternal serum was also not observed, with only IL-2 observed to be significantly decreased in aspirin resistant individuals. However, the sample size based on our inclusion criteria and requirement for complete thromboxane and biomarker measurements is underpowered to detect a significant difference (Post-hoc Power 15.2 %). Further prospective studies powered to study aspirin resistance with a more complete maternal serum biomarker panel are warranted. This would enable assessment of additional possible mechanisms of action of aspirin in pregnancy [42], such as the ability of aspirin to transform COX-2 to the anti-inflammatory compound aspirin triggered 15-epi-lipoxin [59]. Another limitation of this study is the dose of LDA (60 mg daily) is less than that currently recommended (81 mg daily), so what we are defining as aspirin resistance may be a result of a physiologically insufficient dose in some individuals, despite including BMI as a covariate in our statistical models. In addition, this trial did not quantify active salicylic acid metabolites, so we cannot directly discriminate between aspirin resistance and non-adherence.

## 5. Conclusions

This study identifies maternal serum biomarker signatures associated with aspirin resistance in preeclampsia. With 50–85 % of US gravidas eligible to take daily aspirin based on current recommendations [11], appropriate LDA dosage and identifying aspirin resistance are important considerations in clinical care with broad applicability to a large number of individuals in the pregnant population. The results of this study suggest a potential role for IL-2 in the development of preeclampsia modulated by an individual’s response to aspirin. This presents an opportunity to optimize aspirin prophylaxis on an individual level utilizing circulating maternal biomarkers, such as IL-2 and TBX_2_, to quantify an individual’s response to LDA prophylaxis in pregnancy to improve clinical outcomes.

## Supplementary Material

Supplementary Fig. 1. Maternal serum biomarker concentrations of IL-2 at three time points across gestation (randomization, 24–28 weeks, 34–38 weeks) stratified by A. Clinical outcome by LDA group and B. LDA resistant and sensitive groups. FDR = false discovery rate.

Supplementary Fig. 3. Maternal biomarker concentrations of sTNF-R2 at three time points across gestation (randomization, 24–28 weeks, 34–38 weeks) stratified by A. Clinical outcome by LDA group and B. Scatterplot of sTNF-R2 concentration across scaled gestation. FDR = false discovery rate.

Supplementary Fig. 4. Pairwise Pearson correlation coefficients of log2 maternal serum biomarker concentrations. * p < 0.05; ** p <0.01; *** p < 0.001.

Supplementary Fig. 2. Maternal biomarker concentrations of sTNF-R1 at three time points across gestation (randomization, 24–28 weeks, 34–38 weeks) stratified by A. Clinical outcome by LDA group and B. Scatterplot of sTNF-R1 concentration across scaled gestation. FDR = false discovery rate.

## Figures and Tables

**Table 1:** Characteristics of study population stratified by original clinical trial randomization groups and aspirin resistance groups.

Characteristic	Placebo		Low-dose Aspirin (LDA)		p-value	LDA: Healthy			LDA: Preeclampsia			p-value
	Healthy	Preeclampsia	Healthy	Preeclampsia		Sensitive	Resistant	Non-compliant	Sensitive	Resistant	Non-compliant	
**Total N**	**198 (78 %)**	**55 (22 %)**	**227 (84 %)**	**44 (16 %)**	0.12*	**148 (65 %)**	**53 (23 %)**	**26 (12 %)**	**31 (70 %)**	**7 (16 %)**	**6 (14 %)**	**0.42** *
**> 1 pregnancy with PE**					0.8*							**na**
No	54 (27 %)	9 (16 %)	60 (27 %)	12 (27 %)		35 (24 %)	21 (40 %)	4 (15 %)	7 (23 %)	2 (29 %)	3 (50 %)	
Yes	5 (3 %)	2 (4 %)	7 (3 %)	1 (2 %)		2 (1 %)	1 (2 %)	4 (15 %)	1 (3 %)	0	3 (50 %)	
Unknown	139 (70 %)	44 (80 %)	160 (70%)	31 (71 %)		111 (75%)	31 (58 %)	18 (70 %)	23 (74 %)	5 (71 %)	0	
**Hypertension at baseline**	71 (36 %)	19(35 %)	70 (31 %)	19 (43 %)	0.4*	50 (34 %)	14(26 %)	6 (23 %)	15 (48 %)	3 (43 %)	1 (17 %)	**0.3** *
**Protenuric at baseline**	9 (5 %)	5 (9 %)	13 (6 %)	0	0.2*	9 (6.1 %)	0	4 (15 %)	0	0	0	**0.06** *
**Started Hyptertension Medication**					**>0.9** *							**0.5** *
Started prior to pregnancy	39 (20 %)	9 (16 %)	37 (16 %)	11 (25%)		26 (18 %)	9 (17 %)	2 (8 %)	9 (64 %)	2 (100 %)	0	
Started during pregnancy	4 (2 %)	1 (2 %)	6 (3 %)	2 (5 %)		5 (3 %)	0 (0 %)	1 (4 %)	1 (7 %)	0	1 (100 %)	
Did not start	24 (12 %)	4 (7 %)	24 (11 %)	4 (9 %)		17 (11 %)	5 (9 %)	2 (7 %)	4 (29 %)	0	0	
Unknown	131 (66 %)	41 (75 %)	160 (70 %)	27 (61 %)		100 (68 %)	39 (74 %)	21 (81 %)	17 (55 %)	5 (71 %)	5 (83 %)	
**Diabetes**					0.2*							**0.2** *
Yes	34 (17 %)	16 (29 %)	51 (22 %)	7 (16 %)		37 (25 %)	6 (11 %)	8 (31 %)	6 (19 %)	1 (14 %)	0	
No/Unknown	164 (83 %)	39 (71 %)	176 (88 %)	37 (84 %)		111 (75%)	47 (89 %)	18 (69 %)	25 (81 %)	6 (86 %)	6 (100 %)	
**Number of fetuses**					0.8*							**0.5** *
1	160 (80.5 %)	41 (75 %)	185 (81 %)	37 (84 %)		122 (82 %)	42 (79 %)	21 (81 %)	28 (90 %)	5 (71 %)	4 (67 %)	
2	37 (19 %)	14 (25 %)	40 (18 %)	7 (16 %)		25 (17 %)	11 (21 %)	4 (15 %)	3 (10 %)	2 (29 %)	2 (33 %)	
3	1 (0.5 %)	0	2 (1 %)	0		1 (1 %)	0	1 (4 %)	0	0	0	
**BMI**	29 (28,30)	30 (28,33)	29 (28, 30)	30 (28, 32)	**0.4**^+^	29 (28,30)	29 (27,32)	29 (26,31)	29 (27,32)	31 (25,36)	32 (23,42)	**0.8** ^+^
**Maternal Age**	27 (26,28)	28 (26, 29)	26 (26,27)	28 (25,30)	**0.2**^+^	27 (26,28)	23 (22,25)	26 (24,28)	29 (26,31)	27 (21,34)	22 (14,20)	**0.2** ^+^

Data is presented as n (%).

* Pearson’s Chi-squared test; Fisher’s exact test.

+ Kruskal-Wallis rank sum test.

**Table 2:** Maternal biomarker concentrations stratified by aspirin resistance group among pregnancies with normal outcomes.

Biomarker	Timepoint	LDA: Healthy			LDA: Healthy Resistant vs Sensitive	
		Non-adherent, N = 26	Resistant, N = 53	Sensitive, N = 148	p-value^+^	FDR
**IL-2 (pg/mL)**	13–16 weeks	1145 (946, 1343)	970 (890, 1050)	1112 (1,042, 1,182)	0.035	0.112
	24–28 weeks	1004 (847, 1161)	853 (786, 920)	1101 (1016, 1185)	2.80E-04	0.003
	34–38 weeks	971 (841, 1100)	840 (765, 914)	1056 (987, 1125)	4.40E-04	0.003
**IL-6 (pg/mL)**	13–16 weeks	2 (1, 4)	3 (1.5, 3.9)	4 (0.89, 6.9)	1.000	1.000
	24–28 weeks	3 (1.7, 3.8)	2 (1.4, 3.1)	4 (1.6, 6)	0.120	0.251
	34–38 weeks	4 (1.2, 7.3)	4 (2.1, 5.5)	4 (1.5, 7.4)	0.850	0.890
**PLGF (pg/mL)**	13–16 weeks	298 (177, 418)	284 (211, 356)	264 (220, 307)	0.250	0.409
	24–28 weeks	701 (501, 902)	675 (542, 808)	568 (458, 677)	0.041	0.115
	34–38 weeks	497 (364, 630)	573 (330, 817)	375 (291, 459)	0.031	0.112
**sTNF-R1 (pg/mL)**	13–16 weeks	1004 (906, 1102)	868 (808, 927)	957 (904, 1009)	0.061	0.141
	24–28 weeks	1180 (1009, 1352)	976 (908, 1044)	1074 (1020, 1128)	0.038	0.113
	34–38 weeks	1342 (1156, 1528)	1164 (1075, 1252)	1300 (1226, 1374)	0.034	0.112
**STNF-R2 (pg/mL)**	13–16 weeks	2855 (2584, 3126)	2597 (2381, 2813)	2731 (2573, 2889)	0.350	0.508
	24–28 weeks	3209 (2795, 3622)	2630 (2427, 2833)	2953 (2803, 3104)	0.015	0.068
	34–38 weeks	3238 (2815, 3660)	2918 (2644, 3193)	3109 (2948, 3270)	0.170	0.306
**Thromboxane (ng/mL)**	13–16 weeks	19 (13, 24)	29 (24, 35)	19 (16, 22)	4.40E-04	0.003
	24–28 weeks	2 (0.80, 3.0)	31 (24, 37)	1 (0.52, 1.2)	<2e-16	6.00E-15
	34–38 weeks	24 (19, 28)	26 (19, 33)	1 (0.72, 1.5)	1.70E-15	3.83E-14

Data is presented as mean (95% CI).

+ Student’s T-Test – Pairwise Comparison.

**Table 3:** Maternal biomarker concentrations stratified by aspirin resistance group among pregnancies with preeclampsia.

Biomarker	Timepoint	LDA: Preeclampsia			LDA: Preeclampsia Resistant vs Sensitive		LDA: All Samples Resistant vs Sensitive	
		Non-adherent, N = 6	Resistant, N = 7	Sensitive, N = 31	p-value^+^	FDR	p-value^+^	FDR
**IL-2 (pg/mL)**	13–16 weeks	1004 (540, 1,467)	872 (579, 1165)	1098 (912, 1284)	0.240	0.400	0.017	0.073
	24–28 weeks	839 (545, 1132)	830 (638, 1021)	1087 (855, 1320)	0.170	0.306	0.000	0.002
	34–38 weeks	854 (643, 1065)	834 (475, 1194)	1037 (869, 1205)	0.230	0.400	0.000	0.002
**IL-6 (pg/mL)**	13–16 weeks	2 (1.1, 2.9)	2 (1, 2.4)	3 (1.8, 3.6)	0.130	0.254	0.540	0.675
	24–28 weeks	2 (1.6, 2.8)	2 (1, 3.6)	3 (2, 3.4)	0.560	0.690	0.100	0.220
	34–38 weeks	4 (2.2, 5)	3 (1.2, 4.2)	3 (2.7, 4.2)	0.310	0.473	0.740	0.822
**PLGF (pg/mL)**	13–16 weeks	115 (34, 196)	198 (52, 344)	248 (177, 319)	0.510	0.675	0.330	0.487
	24–28 weeks	502 (258, 746)	504 (284, 724)	517 (399, 635)	0.870	0.900	0.057	0.139
	34–38 weeks	610 (68, 1153)	342 (173, 511)	261 (182, 341)	0.130	0.254	0.012	0.057
**sTNF-R1 (pg/mL)**	13–16 weeks	834 (667, 1001)	721 (573, 870)	914 (796, 1031)	0.053	0.133	0.019	0.078
	24–28 weeks	927 (701, 1152)	900 (668, 1132)	978 (841, 1116)	0.650	0.760	0.052	0.133
	34–38 weeks	1327 (1047, 1607)	1195 (734, 1656)	1176 (1030, 1323)	0.980	0.991	0.064	0.144
**sTNF-R2 (pg/mL)**	13–16 weeks	2882 (2153, 3611)	2236 (1650, 2822)	2577 (2292, 2862)	0.290	0.450	0.240	0.400
	24–28 weeks	2842 (1841, 3843)	2469 (1830, 3109)	2785(2292, 2862)	0.600	0.720	0.020	0.078
	34–38 weeks	3435 (2635, 4236)	2838 (2060, 3616)	2741 (2406, 3075)	0.690	0.786	0.290	0.450
**Thromboxane (ng/mL)**	13–16 weeks	24 (−1.3, 50)	19 (6.3, 32)	20 (13, 27)	0.780	0.846	0.001	0.006
	24–28 weeks	2 (−1.7, 5.0)	33 (17, 49)	1 (0.35, 1.7)	2.20E-07	3.96E-06	<2e-16	6.00E-15
	34–38 weeks	36 (13, 60)	15 (−3.8, 34)	1 (0.26, 2.1)	0.039	0.113	<2e-16	6.00E-15

Data is presented as mean (95% CI).

+ Student’s T-Test – Pairwise Comparison.

**Table 4:** Maternal biomarker concentrations among healthy and preeclamptic pregnancies in the LDA and placebo groups.

Biomarker	Timepoint	Placebo		Placebo: Preeclampsia vs Healthy		LDA		LDA: Preeclampsia vs Healthy	
		Healthy, N = 198	Preeclampsia, N = 55	p-value^+^	FDR	Healthy, N = 227	Preeclampsia, N = 44	p-value^+^	FDR
**IL-2 (pg/mL)**	13–16 weeks	1080 (1012, 1148)	1151 (1033, 1269)	0.170	0.306	1083 (1028, 1137)	1049 (905, 1193)	0.450	0.623
	24–28 weeks	968 (908, 1028)	1109 (989, 1228)	0.036	0.112	1032 (971, 1092)	1012 (843, 1181)	0.620	0.734
	34–38 weeks	922 (866, 978)	1052 (936, 1167)	0.036	0.112	996 (945, 1047)	980 (851, 1109)	0.770	0.845
**IL-6 (pg/mL)**	13–16 weeks	2 (1.9, 3)	4 (2, 5.9)	0.044	0.116	3 (2.4, 4.4)	2 (1.8, 3.1)	0.490	0.658
	24–28 weeks	3 (1.7, 4)	4 (2.5, 5)	0.059	0.140	3 (2.6, 4.0)	3 (2.1,3.1)	0.530	0.675
	34–38 weeks	3 (2.8, 3.9)	7 (3.9, 9.1)	0.005	0.028	4 (3.3, 5.3)	3 (2.8, 3.9)	0.790	0.846
**PLGF (pg/mL)**	13–16 weeks	320 (265, 375)	249 (198, 300)	0.920	0.941	272 (237, 308)	222 (167, 277)	0.470	0.641
	24–28 weeks	630 (560, 701)	468 (366, 570)	0.009	0.048	608 (527, 689)	513 (423, 602)	0.600	0.720
	34–38 weeks	440 (367, 512)	262 (198, 326)	0.003	0.018	435 (355, 515)	322 (234, 409)	0.320	0.480
**sTNF-R1 (pg/mL)**	13–16 weeks	955 (903, 1006)	1008 (897, 1119)	0.400	0.571	941 (903, 980)	872 (785, 960)	0.110	0.236
	24–28 weeks	1007 (968, 1046)	1146 (1041, 1251)	0.011	0.055	1063 (1020, 1106)	959 (857, 1061)	0.030	0.112
	34–38 weeks	1196 (1146, 1247)	1437 (1297, 1576)	0.001	0.006	1273 (1216, 1329)	1200 (1079, 1320)	0.240	0.400
**sTNF-R2 (pg/mL)**	13–16 weeks	2661 (2509, 2812)	2941 (2680, 3201)	0.042	0.115	2714 (2596, 2832)	2564 (2336, 2793)	0.290	0.450
	24–28 weeks	2671 (2554, 2789)	3187 (2929, 3444)	0.000	0.002	2907 (2788, 3026)	2743 (2341, 3144)	0.130	0.254
	34–38 weeks	2801 (2681, 2921)	3374 (3077, 3670)	0.000	0.003	3079 (2949, 3210)	2851 (2579, 3123)	0.150	0.287
**Thromboxane (ng/mL)**	13–16 weeks	18 (15, 20)	18 (14, 21)	0.710	0.799	21 (19, 24)	20 (15, 26)	0.450	0.623
	24–28 weeks	19 (16, 21)	18 (13, 22)	0.680	0.785	8 (5.7, 10)	6 (2.1, 10)	0.540	0.675
	34–38 weeks	18 (16, 21)	18 (13, 24)	0.810	0.858	9 (7.2, 12)	8 (3.2, 13)	0.520	0.675

Data is presented as mean (95% CI).

+ Student’s T-Test – Pairwise Comparison.
