# Supplementary figures and images for: Aspirin resistance in pregnancy is associated with reduced interleukin-2 (IL-2) concentrations in maternal serum: Implications for aspirin prophylaxis for preeclampsia
Source: Pregnancy Hypertens. Author manuscript; Available in PMC 2024 Dec 2. (PMC11610477; doi:10.1016/j.preghy.2024.101131)

**A.**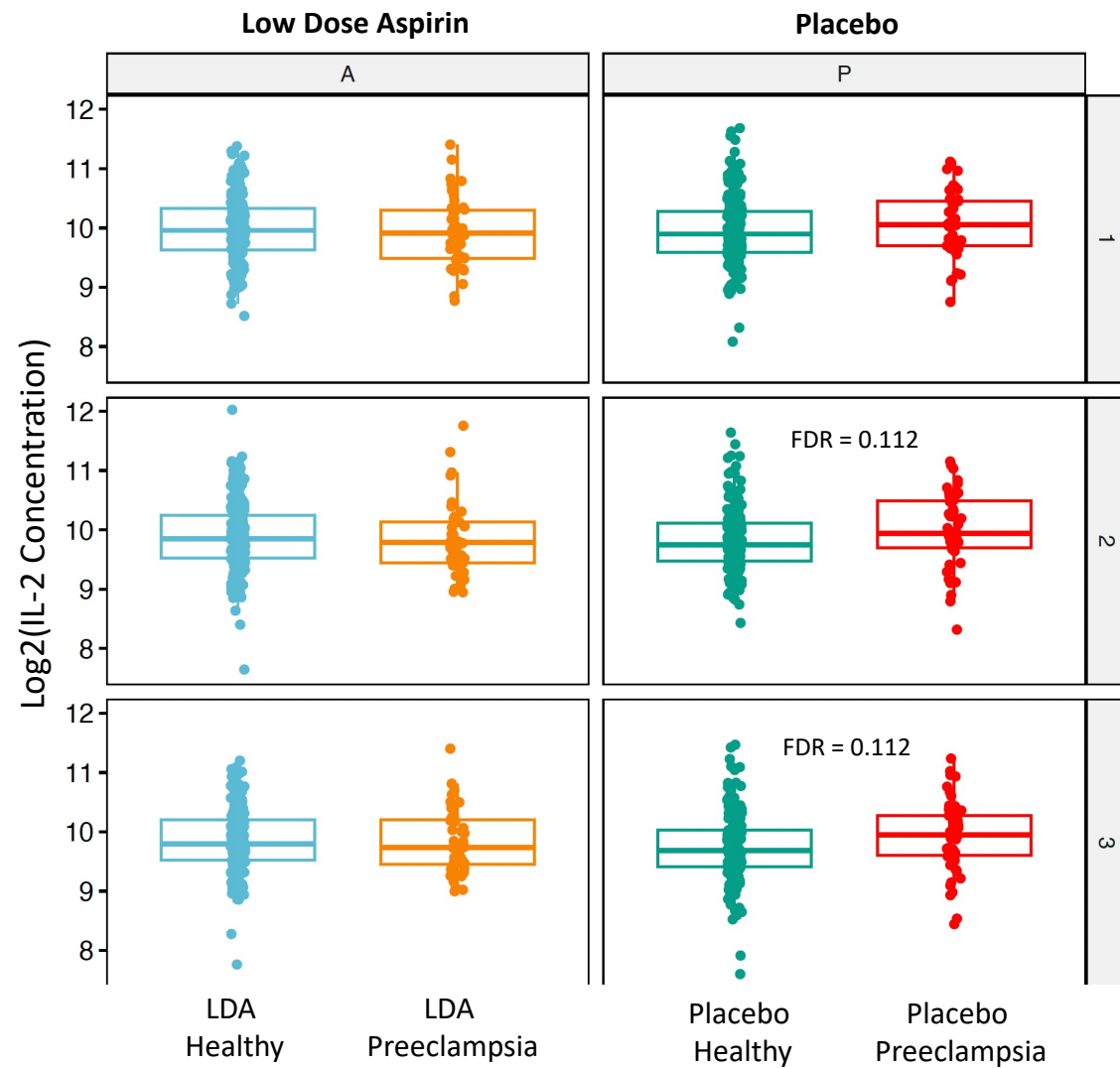**B.**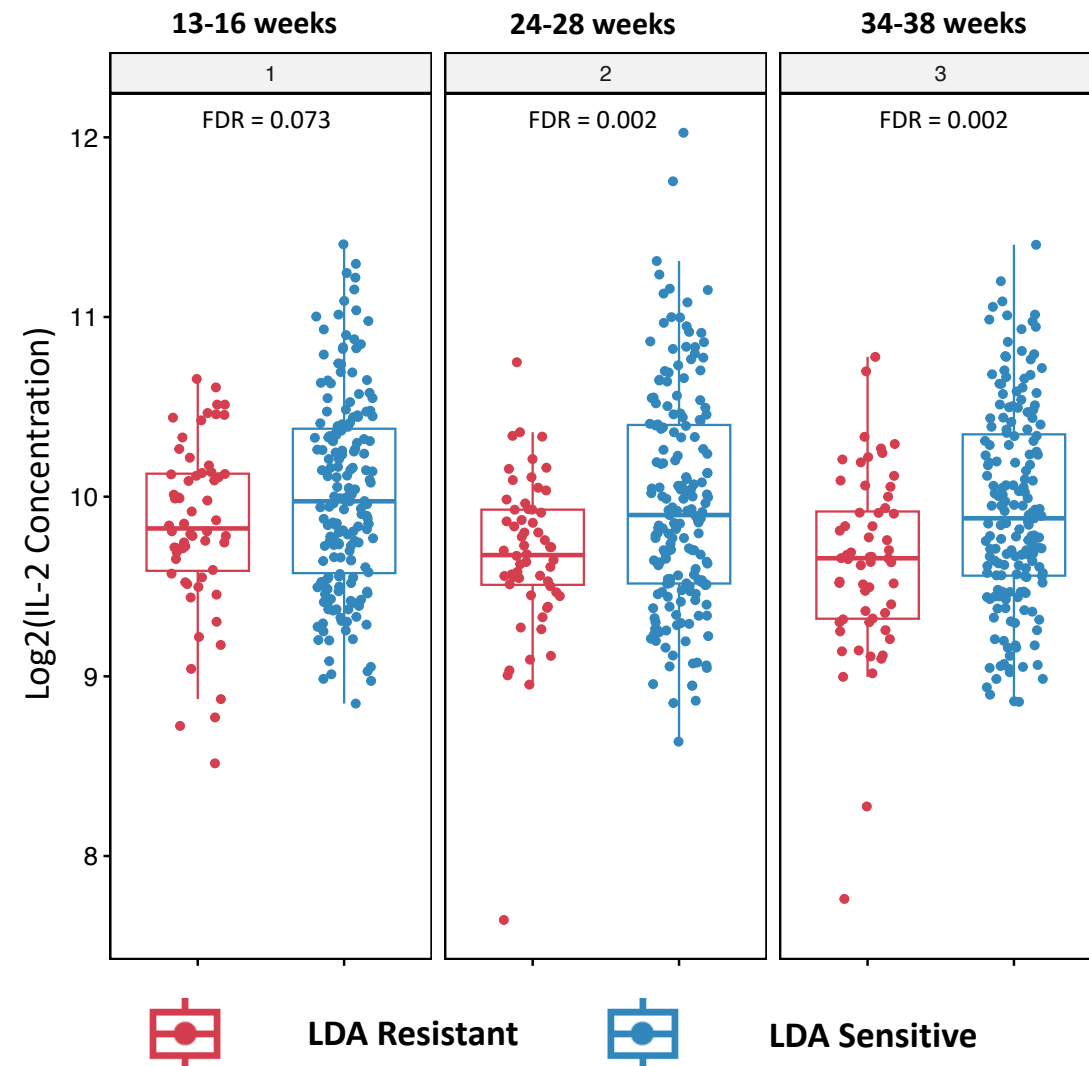

Supplement: Supplementary Fig. 1. Maternal serum biomarker concentrations of IL-2 at three time points across gestation (randomization, 24–28 weeks, 34–38 weeks) stratified by A. Clinical outcome by LDA group and B. LDA resistant and sensitive groups. FDR = false discovery rate. [file NIHMS2029825-supplement-Supplementary_Fig__1__Maternal_serum_biomarker_concentrations_of_IL-2_at_three_time_points_across_gestation__randomization__24_28_weeks__34_38_weeks__stratified_by_A__Clinical_outcome_by_LDA_group_and_B__LDA_resistant_.pdf]

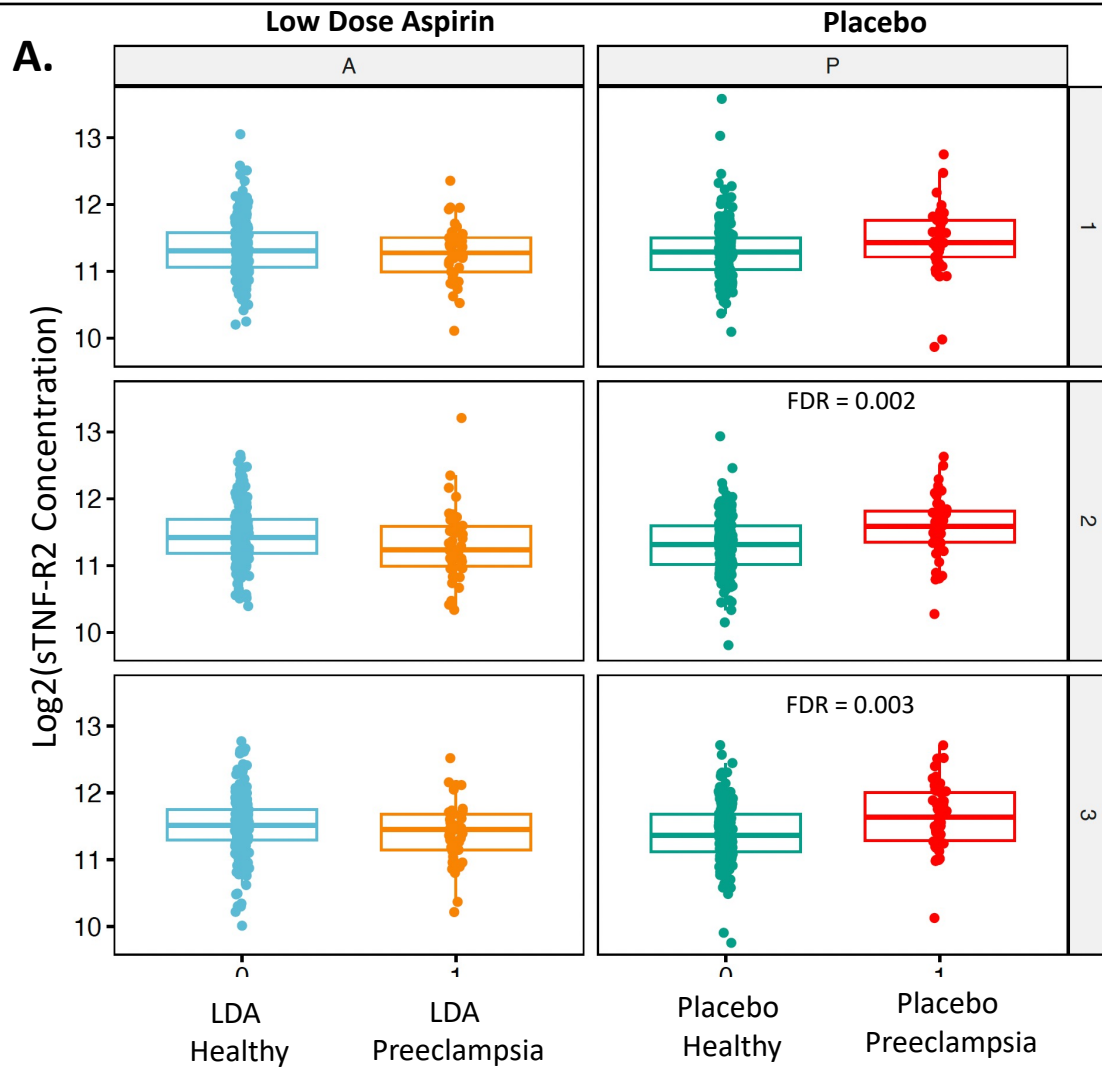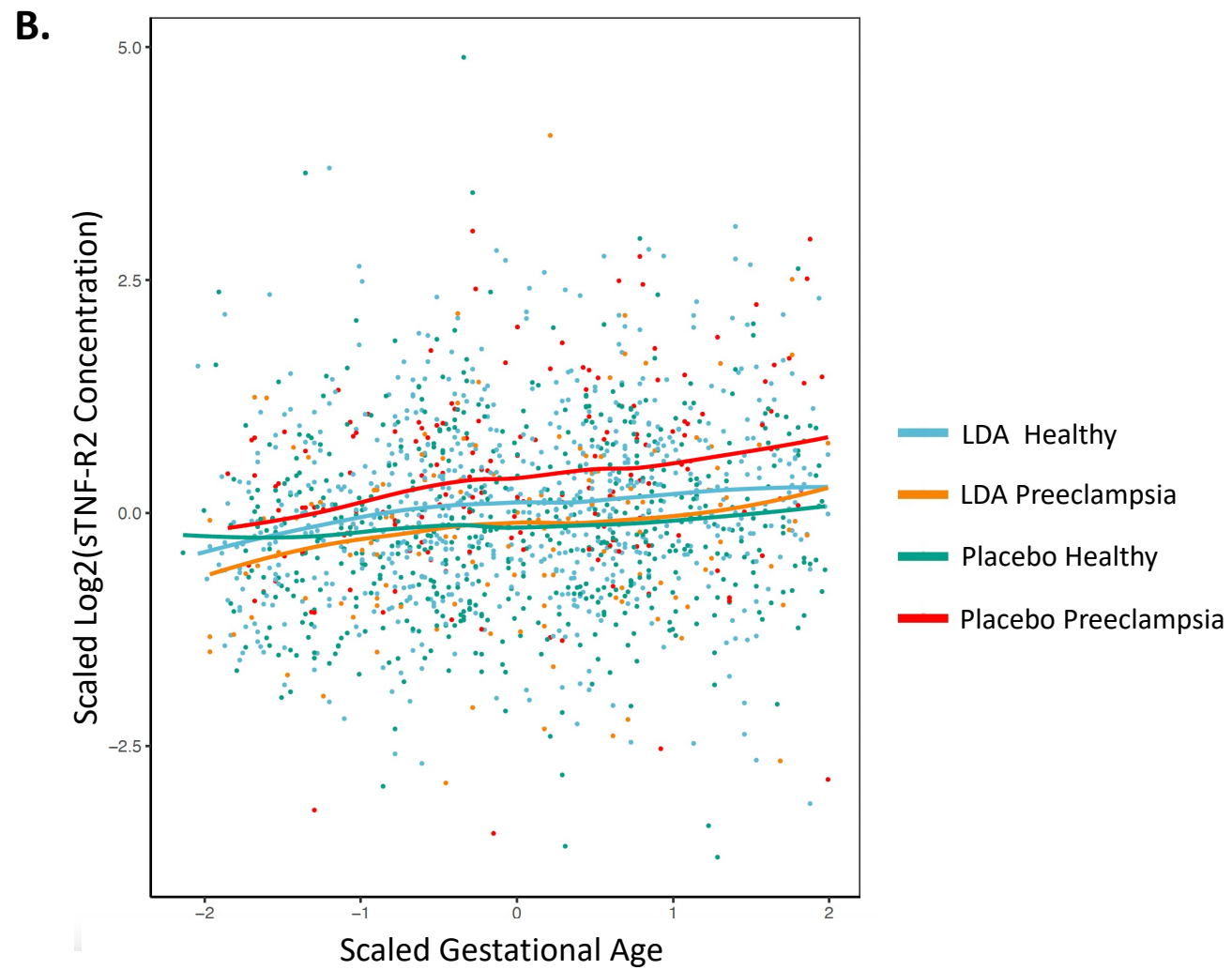

Supplement: Supplementary Fig. 3. Maternal biomarker concentrations of sTNF-R2 at three time points across gestation (randomization, 24–28 weeks, 34–38 weeks) stratified by A. Clinical outcome by LDA group and B. Scatterplot of sTNF-R2 concentration across scaled gestation. FDR = false discovery rate. [file NIHMS2029825-supplement-Supplementary_Fig__3__Maternal_biomarker_concentrations_of_sTNF-R2_at_three_time_points_across_gestation__randomization__24_28_weeks__34_38_weeks__stratified_by_A__Clinical_outcome_by_LDA_group_and_B__Scatterplot_of_sT.pdf]

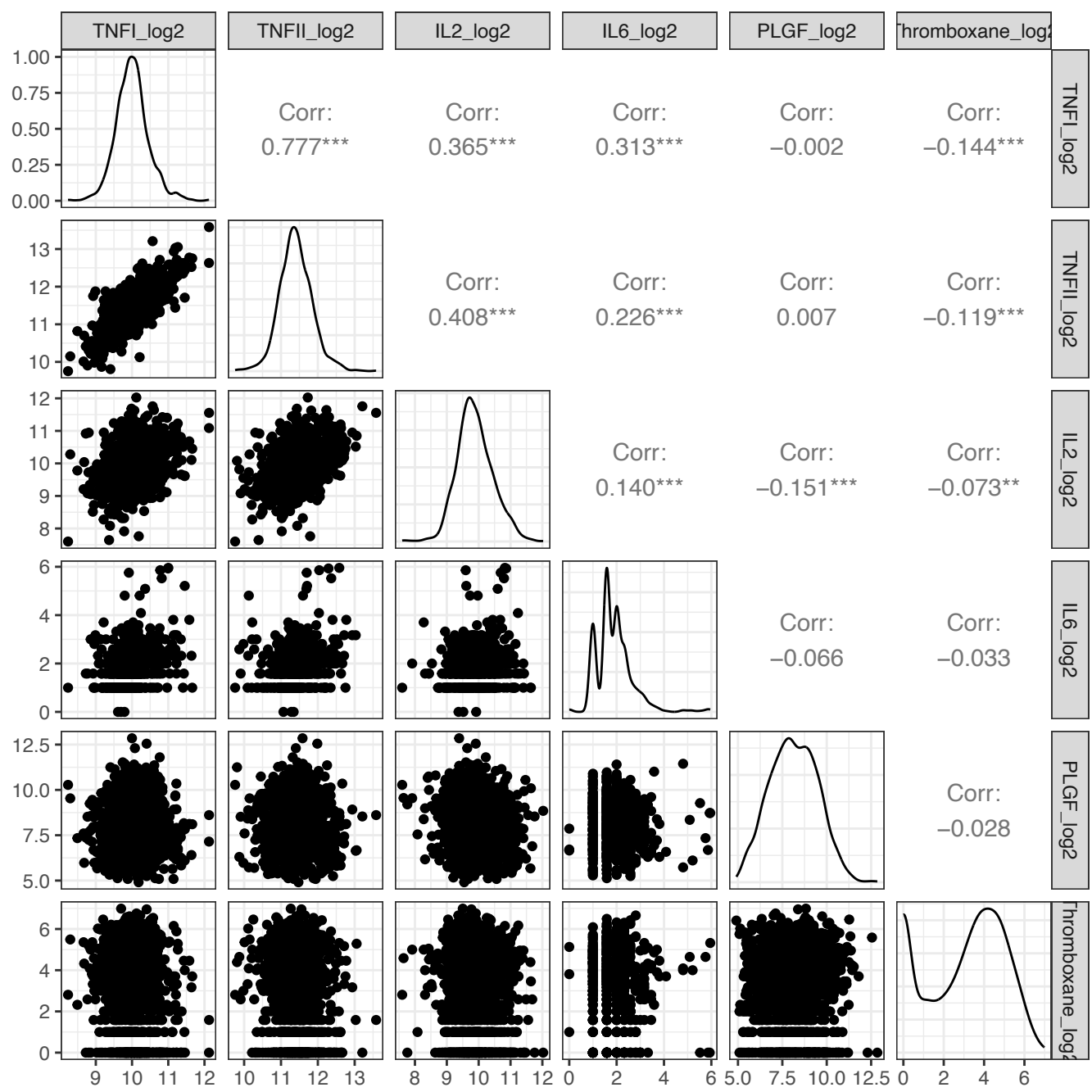

Supplement: Supplementary Fig. 4. Pairwise Pearson correlation coefficients of log2 maternal serum biomarker concentrations. * p < 0.05; ** p <0.01; *** p < 0.001. [file NIHMS2029825-supplement-Supplementary_Fig__4__Pairwise_Pearson_correlation_coefficients_of_log2_maternal_serum_biomarker_concentrations____p___0_05_____p__0_01______p___0_001_.pdf]

**A.**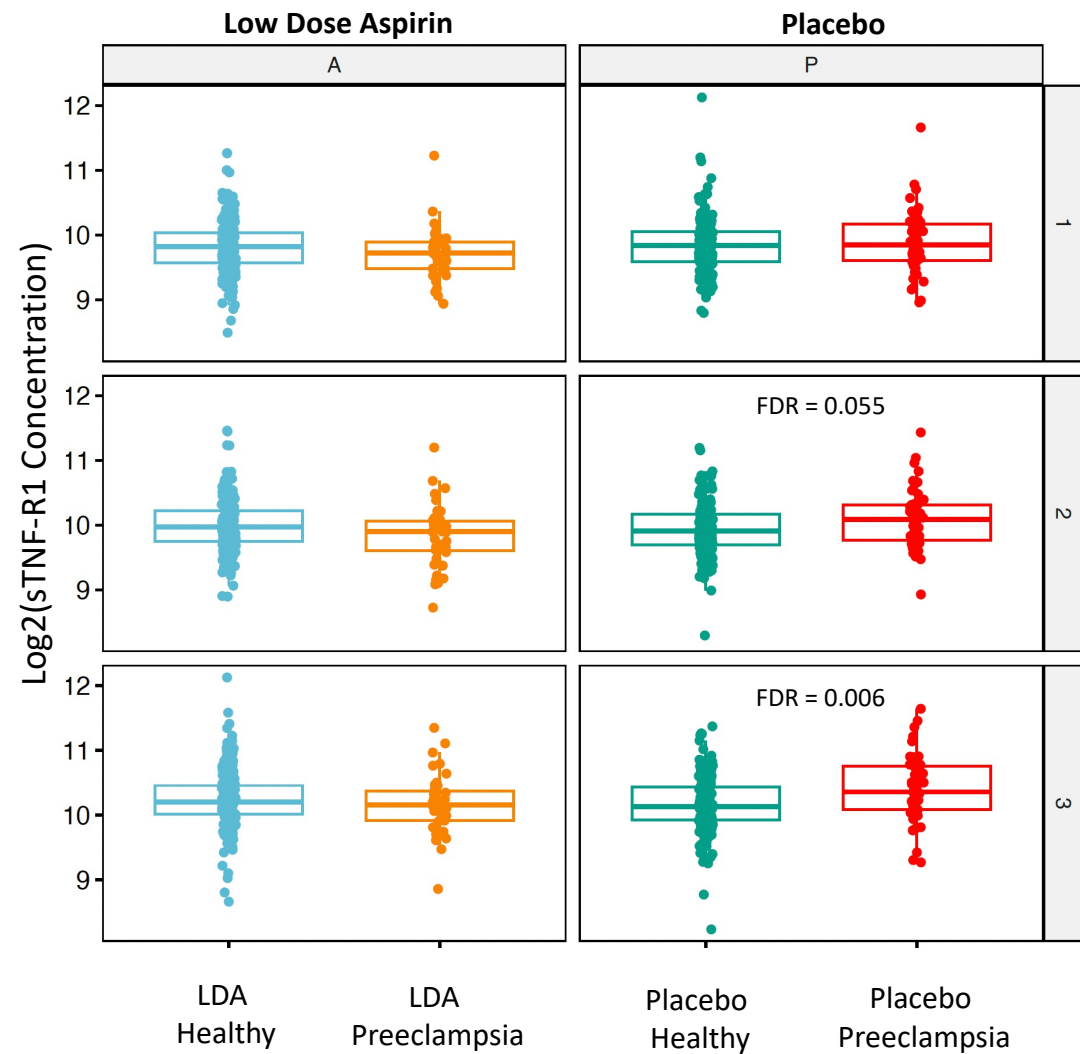**B.**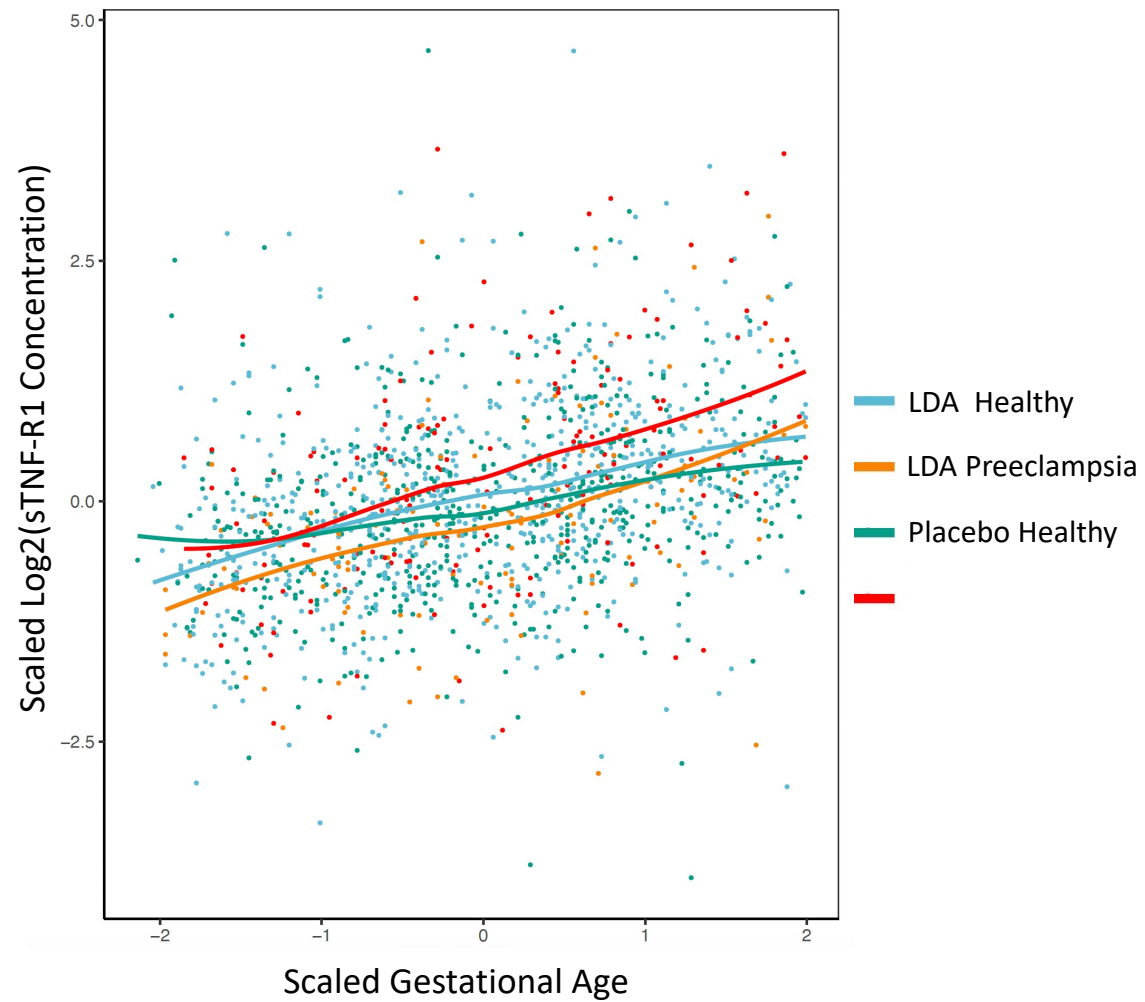

Supplement: Supplementary Fig. 2. Maternal biomarker concentrations of sTNF-R1 at three time points across gestation (randomization, 24–28 weeks, 34–38 weeks) stratified by A. Clinical outcome by LDA group and B. Scatterplot of sTNF-R1 concentration across scaled gestation. FDR = false discovery rate. [file NIHMS2029825-supplement-Supplementary_Fig__2__Maternal_biomarker_concentrations_of_sTNF-R1_at_three_time_points_across_gestation__randomization__24_28_weeks__34_38_weeks__stratified_by_A__Clinical_outcome_by_LDA_group_and_B__Scatterplot_of_sT.pdf]
